# Prevalence of Dermatophytosis and Antifungal Activity of Ethanolic Crude Leaf Extract of* Tetradenia riparia* against Dermatophytes Isolated from Patients Attending Kampala International University Teaching Hospital, Uganda

**DOI:** 10.1155/2019/9328621

**Published:** 2019-07-11

**Authors:** Taufik Kakande, Yonah Batunge, Emmanuel Eilu, Ambrose Shabohurira, Justus Abimana, Saheed Adekunle Akinola, Reagan Muhwezi, Abubakar Sunusi Adam, Sarah Kemuma Onkoba, Adamu Almustapha Aliero, Collins Atuheire, Charles Drago Kato, Ibrahim Ntulume

**Affiliations:** ^1^School of Pharmacy, Kampala International University Western Campus, Ishaka, P.O. Box 71, Bushenyi, Uganda; ^2^Department of Medical Laboratory Science, Kampala International University Western Campus, Ishaka, P.O. Box 71, Bushenyi, Uganda; ^3^Department of Microbiology and Immunology, Faculty of Biomedical Sciences, Kampala International University Western Campus, Ishaka, P.O. Box 71, Bushenyi, Uganda; ^4^Department of Public Health, Kampala International University Teaching Hospital, P.O. Box 71, Bushenyi, Uganda; ^5^School of Bio-Security, Biotechnical and Laboratory Sciences, College of Veterinary Medicine, Animal Resources and Biosecurity (COVAB), Makerere University, P.O. Box 7062, Kampala, Uganda

## Abstract

Dermatophyte infections are a global health problem but neglected in Uganda. This work aimed at determining prevalence of dermatophytosis and antifungal activity of ethanolic crude leaf extract of* Tetradenia riparia* against dermatophytes isolated from patients attending Kampala International University Teaching Hospital (KIU-TH), Uganda. A total of 100 samples of skin and nail scrapings were collected and processed using standard microscopy (KOH) and cultural methods.* T. riparia* leaves were collected and processed with 95% ethanol using standard extraction method. The crude leaves ethanolic extract was tested against three dermatophytes:* Trichophyton tonsurans*,* T. mentagrophyte, *and* Microsporum audouinii* using modified agar well diffusion method. Minimum inhibitory concentration (MIC) and minimum fungicidal concentration (MFC) of the ethanolic leaves crude extract were also determined using broth tube dilution and culture, respectively. Out of 100 samples collected, 49 (49%, 95%CI: 0.3930-0.5876) were found positive for microscopy. The prevalence of dermatophytosis was significantly (p=0.001) associated with age groups of participants with higher infection among those aged 11-20 and 21-30 years with 75.0% each. Out of the 49 that were positive by microscopy, 28 (57.15%, 95% CI: 0.1987-0.3739) were positive by culture. Thirty-one (31) fungal isolates were obtained which included both dermatophyte and non-dermatophyte fungi.* T. verrucosum* had highest distribution 6 (19.35%) among dermatophytes species while* Aspergillus* spp. were found to have highest distribution 7 (22.58%) among non-dermatophyte species. The result of the antidermatophytic test showed that* T. riparia *ethanolic crude leaves extract had activity against tested dermatophytes at 1 g/ml. MIC and MFC of the crude extract of* T. riparia *against tested dermatophytes ranged from 62.5 to 250 mg/ml and 125 to 500 mg/ml, respectively. The findings of this study reported the presence of dermatophytes causing dermatophytosis among patients attending KIU-TH. The results of the current study showed that* T. riparia *leaves ethanolic crude extract has antidermatophytic activity against tested dermatophytes.

## 1. Introduction

Dermatophytes are a group of fungi that invade keratinized tissues of the hair, skin (epidermis), and nails, and some cause conditions commonly referred to as Tinea-meaning group of disease. They grow best in humid and warm environment hence common in the subtropical and humid regions. Recent classifications of dermatophytes using multilocus phylogenetic study regroup the organisms into seven (7) genera:* Arthroderma, Epidermophyton, Lophophyton, Microsporum, Nannizzia, Paraphyton, *and* Trichophyton* [1]. The most causative organisms of tinea capitis fall in three genera:* Trichophyton, Microsporum*, and Nannizzia [1].* Tinea *infection can spread from person to person (anthropophilic) or indirectly from fomites (e.g., clothes hair brush and huts), through soil (geophilic) or contact with animals (zoophilic) [2]. They infect areas of the body such as the groin (*Tinea cruris* or “jock itch”), hands/legs, full body (*Tinea corporis* or “ringworm”), hips, waist, scalp (*Tinea capitis* or “scalp ringworm”), and nails (*Tinea unguium * or onychomycosis), respectively [2–4]. As reviewed by Bongomin et al. [5], the global burden of cutaneous infection was estimated to be ~1001000, 000. These infections are more common among rural than urban population; the infections are more pronounced in males as compared to females and high in children especially* Tinea capitis* [6]. Literature has shown that the global burden of dermatophytic infection specifically was estimated to be 20–25% [7]. There was scarcity of data on dermatophytic infection in Uganda especially from western part of the country, but* T. capitis* affects 10% of all Kenyan school children [7, 8]. Wiegand et al. [9] reported 82.6% prevalence of dermatophytosis in children attending Mbarara Regional Referral Hospital in Uganda. Dermatophyte infections are therefore a serious but neglected health problem in Uganda [10]. Several species of* Trichophyton* and* Microsporum* have been isolated from tinea capitis (“scalp ringworm”) lesions [10]. The predominant infecting species is* T. tonsurans*. Disease manifestations range from small scaling patches to involvement of the entire scalp with extreme hair loss [10]. Although* T. capitis* infections are not life threatening, they have significant social and health related economic impacts [5, 11] due to reoccurrences from exposure to the sources of the causative organisms such as sharing towels, clothing, or hair accessories with infected individuals [8], long duration of treatment regimens, poor adherence, and cases of reoccurrences [12].

Although many effective antifungal drugs are available, most of them are relatively expensive [13] and others with marked toxic effects such as hepatotoxicity. Plants used in herbal treatment as antifungal agents such as* T. riparia* [14] could provide an alternative, relatively cheap, and improved accessibility to treatment for such opportunistic infections especially in HIV/AIDS.

*T. riparia* or misty plum bush (English) is a medicinal plant also locally known as kyewamala (Luganda), Akachuchu (Rotoro), and Omuravunga (Runyankole). It is used locally for the treatment of a variety of illnesses such as malaria, several skin diseases (fungi, cleaning wounds, itching, body rashes, and scabies), ulcers, headache, bilharzias, worms, rheumatism, emetic, dyspepsia, fever, colds, cough, toothache, psychotic excitements, and blocked fallopian tubes [15]. In Uganda,* T. riparia *plant was reported to be used in treatment of both bacterial and fungal infection [14]. Therefore, this study was aimed at determining the prevalence of dermatophytosis and antifungal activity of ethanolic crude leaf extract of* T. riparia* against dermatophytes isolated from patients attending Kampala International University Teaching Hospital, Uganda.

## 2. Materials and Methods

### 2.1. Study Area and Study Design

This was a laboratory experimental study involving isolation and identification of dermatophytes causing fungi from patients attending Community HIV-AIDS Initiative (CHAI) and Dermatology Clinic at Kampala International University Teaching Hospital, Western Campus, Uganda. Isolation and identification of dermatophytosis causing fungi were done at Mycology Laboratory, Department of Microbiology, Mycology Unit, Mbarara Regional Referral Hospital. Plant samples (leaves) were collected from Rukararwe Bushenyi District (0°31′27.0′′S 30°13′02.1′′E) [16]. Extraction, phytochemical screening, and antidermatophytic activity testing were carried out at the Microbiology and Immunology and Pharmacology Laboratories at Department of Microbiology and Immunology and School of Pharmacy Kampala International University, Western Campus.

### 2.2. Inclusion and Exclusion Criteria

All patients that attended CHAI and Dermatology Clinic of KIU-TH presenting with skin diseases, who consented or their parents assented on their behalf in case of patients below 18 years old, were included in this study. Patients that attended CHAI and Dermatology Clinic of KIU-TH presenting with signs and symptoms of dermatophytosis and did not consent were excluded. Patients who were on antifungal treatment were not also included in this study.

### 2.3. Sample Collection

One hundred (100) samples of infected skin, nail, and scalp craping were collected from selected study participants attending Community HIV-AIDS Initiative (CHAI) and Dermatology Clinic through the help of a volunteer who is registered health personnel (medical laboratory personnel). Samples were collected according to the method described by Taha et al. [17] with little modification. The sites of infections were first cleaned with surgical spirit, and scales from the skin lesions were collected by scraping outwards with a blunt scalpel from the edge of the lesion. Specimens from the scalp and nail were collected using forceps by plucking the specimen from the scalp and nail. All samples were collected on a sterile piece of paper (5 cm square). The papers were folded to enclose the specimen, labeled, and transferred to Mycology Laboratory, Department of Microbiology, Mycology Unit, Mbarara Regional Referral Hospital, for isolation and identification.

### 2.4. Isolation and Identification of Tests Organisms

Samples collected were subjected to direct microscopic examination after immersing them in 10% KOH for skin scrapings or hair for ten minutes and 20% KOH for nail for twenty minutes and viewed under microscope using X10 lower magnification and confirmed using X40 higher magnification for the presence of hyphae and arthroconidia, respectively [18]. Scrapings of scalp nail and skin samples were inoculated on the freshly prepared Sabourad Dextrose Agar (SDA) containing chloramphenicol at a concentration of 0.5 mg/ml and 0.4 g/l of cycloheximide in order to inhibit the growth of bacteria and saprophytic fungi, respectively. The inoculated plates were incubated at 25°C for up to 21 days. Suspected colony of dermatophytes was subcultured to freshly prepared SDA media to obtain pure cultures. The suspected colonies of dermatophytes and molds were identified to species level based on macroscopy (colony color, pigment production, topography, and texture) and microscopic morphology such as small unicellular microconidia and larger septate macroconidia using Lactophenol cotton blue mount [19].* Candida* spp. were identified also using phenotypic methods of chromogenic media and germ tube test.

### 2.5. Plant Samples Collection

Plant sample collection, identification, and drying were done according to the method described by Esazah [14]. The fresh leaves of* T. riparia* were collected from Rukararwe, Bushenyi District. The plants materials were taken for identification to a Botanist at Mbarara University of Science and Technology. The plants materials were identified using Martin the Kew database available at www.theplantlist.org [14]. The leaves were dried under shade to avoid decomposition of volatile chemical constituents. The dried leaves were powdered using mortar and pestle, then sieved into fine powder, and stored in airtight jar.

### 2.6. Extraction of Ethanolic Crude Extract

Extraction of the ethanolic crude extract was carried out according the method described by Esazah [14] with some modification. Two hundred grams (200 g) of powder was soaked in ethanol (95% v/v) in a two-liter (2 l) conical flask, with periodic shaking for 24 h. The extract was filtered off using Whatman No. 1 filter paper and concentrated (ethanol left to evaporate off) in hot air oven at 45°C. The concentrated crude extracts were kept at 4°C in a refrigerator in a container and labelled appropriately. The yield (%, w/w) from dried leaves crude extract was calculated using the following formula: yield (%) = (W_1_ x 100)/W_2_, where W_2_ is the weight of powder before drying and W_1_ is the weight of dry extract.

### 2.7. Phytochemical Screening

Phytochemical analysis of leaves crude ethanolic extract of* T. riparia* was done using standard procedures [20–22] with minor modification [23]. The parameters determined are tannins, phlobatannins, saponins, flavanoids, terpenoids, diterpenes, glycosides, alkaloids, steroids, and phenols.

### 2.8. Testing for Antidermatophytic Activity

The test organisms:* T. tonsurans, T. mentagrophyte*, and* M. audouinii* were aseptically inoculated at the center in different plates on freshly prepared sterile Sabouraud Dextrose Agar. Using sterile glass cork borers (6mm in diameter), 3 wells were carefully made on the agar plates without distorting the media at 0.05cm distance away from the inoculated test organism. Two grams (2 g) of* T*.* riparia* leaves crude extract was dissolved in 2 ml of DMSO as stock solution [14]. Each well was filled with 100 *μ*l of 1g/ml extract and the other wells were filled with terbinafine 40 *μ*g/ml as positive control and 10% DMSO as negative control, respectively [22]. The plates were then incubated at 27-30°C for 3-5 days [20]. The antidermatophytic activity was observed using dissecting microscope.

### 2.9. Minimum Inhibitory Concentration (MIC)

*T. tonsurans, T. mentagrophyte*, and* M. audouinii* sporangial suspension concentration was adjusted to an optical density of 0.014 equivalent to 1.0x10^5^ spores/ml using a spectrophotometer at a wave length of 530 nm [20]. MIC* of T. riparia *leaves ethanolic crude extract was determined using tube dilution method. One milliliter (1ml) of Brain Heart infusion Broth + 0.5% Agar was placed in 10 tubes. One milliliter (1 ml) of stock solution 2 g/ml was transferred in the first test tube of each organism mixed and 2-fold serial dilution was done till concentrations below 0.0156 g/ml were attained [14, 23]. One milliliter (1ml) of positive control terbinafine (2 mg/ml) was transferred in different set of test tubes and also serially diluted (2-fold) using the same procedure as above. Then twenty microlitres (20*μ*l) of inoculum preparation of each fungus was placed in the above serially dilluted tubes. One milliliter (1 ml) of 10% DMSO was used as negative control. The test tubes were then incubated at 28 to 30°C for 4-7days. The test tubes were observed for the lowest concentration with no visible growth (no visual turbidity) and were considered as the MIC [23].

### 2.10. Determination of Minimum Fungicidal Concentration

Following the MIC determination using tube dilution method, MFC was determined by subculturing 20*μ*l of the culture from each negative well (with no visual turbidity) of both the extract and the positive control. MFC was defined as the lowest concentration resulting in negative subcultures (no growth) after incubation of the plates at 28 to 30°C for 3-5 days [20].

### 2.11. Ethical Consideration

Ethical approval was sought from the Kampala International University Research and Ethics Committee (KIU-REC). Permission to carry out the study was sought from the School of Allied Health Sciences, KIU-Western Campus. Participation in the study was on voluntary basis. Patients were enrolled in the study after consenting to the study following a verbal and written explanation of the study. For children, consent was provided by parents/legal guidance but assent was also requested from the children. All information was treated with utmost confidentiality.

### 2.12. Data Analysis

Results obtained were entered in Microsoft excel sheet. Descriptive analysis was used to obtain prevalence of dermatophytosis, mean and standard deviation of the age groups of the studied participants. Chi-square was used to compare between the prevalence of dermatophytosis and demographic characteristics of studied participants using SPSS software (version 16) and p≤ 0.05 was considered significant.

## 3. Results

### 3.1. Demographic Characteristic of Studied Participants

One hundred (100) patients selected from patients attending CHAI and Dermatology Clinic of KIU-TH were enrolled in this study. Fifty-three (53%) were males and 47 (47%) were females. The age of the studied participants ranged from 1 to 69 years. The median age of study participants was 34.7 years.

### 3.2. Prevalence of Dermatophytosis

Out of 100 specimens collected, 49 (49 %, 95% CI: 0.3930-0.5876) were KOH positive. The prevalence of dermatophytosis based on microscopy according to gender showed that male participants had the highest prevalence 26/49 (53.1%) compared to their female counterpart 23/49 (46.9%). The gender of the participants was not significantly associated with prevalence of dermatophytosis with microscopic examination using chi-square test (p=0.990) (Table 1). The prevalence of dermatophytosis among the studied participants according to their age groups based on microscopy showed that age groups of 11-20 and 21-30 years had the highest prevalence with 75.0% each while age groups between 51 and 60 and >60 had the lowest prevalence with 10.00 % each. However, the age group of the participants was statistically associated with the prevalence of dermatophytosis with microscopic examination using chi-square tests (p= 0.001) (Table 2).

Out of the 49 samples that were KOH positive, 28 (57.14%, 95%CI: 0.1987-0.3739) were culture positive. Prevalence of dermatophytosis based on culture showed that male had the highest prevalence 19/49 (38.78%) as in Table 1. The prevalence of dermatophytosis based on culture according to the age group of studied participants showed that age groups between 11 and 20 had the highest prevalence 10/49 (20.41%) while age groups between 51 and 60 and >60 each had the lowest prevalence 0/49 (0.00%) as in Table 2.

### 3.3. Distribution of Dermatophytes and Non-Dermatophytes

**Figure 1:**
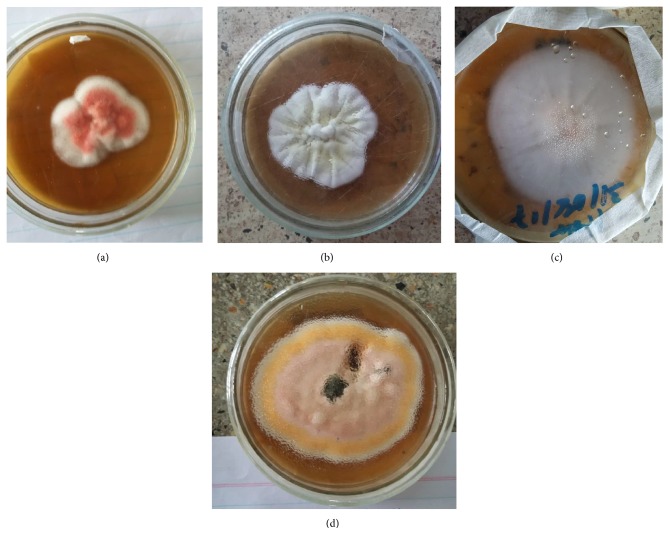
Colony morphology of some isolated dermatophytes species from patients attending Kampala International University Teaching Hospital, Uganda. (a)* T. rubrum, *(b)* T. mentagrophytes, *(c)* M. audouinii*, and (d)* T. tonsurans.*

All fungal isolates obtained were identified using standard colony morphological and microscopic characteristics (Table 3). Thirty-one fungal isolates were isolated from the 28 positive culture samples which included both dermatophytes (Figure 1) and non-dermatophytes fungi. Among the dermatophytes species,* T. verrucosum* was found to have the highest distribution 6 (19.35) in both gender and age of studied participants, while Aspergillus spp. were more distributed 7 (22.58) among non-dermatophytes species in both gender and age of studied participants as in Table 4.

### 3.4. Percentage Yield and Qualitative Phytochemical Screening of* T. riparia* Ethanolic Crude Leaves Extract

The* T. riparia* ethanolic crude extract yielded 20.1 g of powder upon concentration giving a percentage yield of 10.05%. The quantitative phytochemical screening of the* T. riparia* ethanolic leaves crude extract studied showed the presence of tannins, phenols, glycosides, and terpenoids and absence of saponins, flavanoids, phlobatannins, and anthraquinone (Table 5).

### 3.5. Antidermatophytic Susceptibility Assay of* T. riparia* Ethanolic Leaves Crude Extract

**Figure 2:**
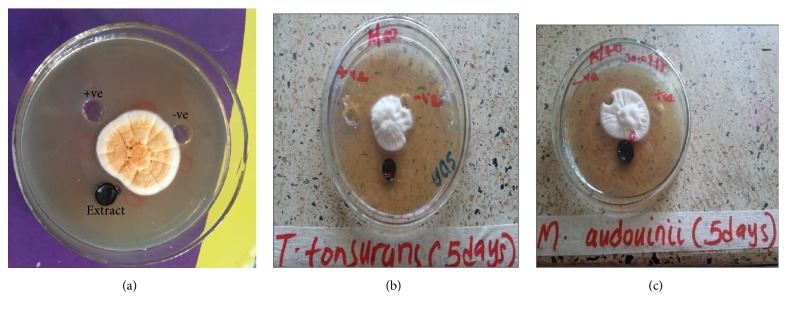
An antidermatophytic activity of* Tetradenia riparia* leaves crude extract, Terbinafine and %10 DMSO against dermatophytes. (a)* T. mentagrophyte*, (b)* T. tonsurans*, and (c)* M. audouinii*. +ve: positive control, -ve: negative control.

The antidermatophytic assay showed that* T. riparia* leaves crude extract and terbinafine (positive control) were active on all test dermatophytes (*T. tonsurans, T. mentagrophyte*, and* M. audouinii*) studied at a concentration of 1g/ml and 40*μ*g/ml, respectively. The negative control (10% DMSO) showed no antidermatophytic activity (Figure 2). However, susceptibility of both terbinafine and extract varied among organisms with* T. tonsurans* being more susceptible followed by* T. mentagrophyte* while* M. audouinii *was less susceptible. Furthermore, terbinafine which was used as positive control showed higher antidermatophytic activity against all test dermatophytes compared to* T. riparia* leaves crude extract.

### 3.6. Minimum Inhibitory Concentration and Minimum Fungicidal Concentration

The MIC of the* T. riparia* leaves crude extract ranged from 62.5 to 250 mg/ml.* T. tonsurans* had 62.5 mg/ml MIC, 125 mg/ml for* T. mentagrophyte*, and 250 mg/ml for* M. audouinii*. Terbinafine as positive control was effective at concentration of 0.0078 mg/ml against* T. tonsurans*, and 0.0078 and 0.0313 mg/ml against* T. mentagrophyte* and* M. audouinii,* respectively (Table 6).

The MFC value of the* T. riparia* leaves crude extract against all tested dermatophytes spp. ranged from 125 to 500 mg/ml. The MFC of the extract against* T. tonsurans* was 125 mg/ml, 250 mg/ml for* T. mentagrophyte*, and 500 mg/ml for* M. audouinii*. MFC value of terbinafine ranged from 0.0156 to 0.0625 mg/ml. Terbinafine was found to have MFC at concentration of 0.0156 mg/ml against* T. tonsurans* and 0.0313 mg/ml and 0.0625 mg/l against* T. mentagrophyte* and* M. audouinii,* respectively. The low MFC values as those obtained with* T. tonsurans *and* T. mentagrophyte* denoted that these dermatophytes spp. are more susceptible to the antifungal agent while a high MFC value observed against* M. audouinii* denoted that this dermatophyte sp. was less susceptible to the antifungal agent (Table 6).

## 4. Discussion

Literatures have shown that fungal infections of the skin and scalp (dermatophytosis) represent a relatively common problem especially in the tropical and subtropical regions of the world where warm and humid climate provides a favourable environment for fungi [5, 24–26]. They have become a significant health problem affecting children, adolescents, and adults [27–29].

The result of the present study found the prevalence of dermatophytosis 49% among patients attending CHAI and Dermatology Clinic at KIU-HT using microscopy (KOH). The prevalence found in this study was lower compared to the prevalence 82.6% (n=115) reported by Wiegand et al. [9] in children attending Mbarara Regional Referral Hospital in Uganda using Blankophor. The lower prevalence found in this study compared to Wiegand et al. [9] could be due to differences in studied participants where we used age groups between 1-67 years while in their study studied participants age groups were 1-16 years. Literature shows that dermatophytosis affects young children than the old [5, 6, 25]. However, Moto et al. [8] reported prevalence of 68.0% (n=150) in school going children from Mathare, informal settlement in Nairobi, Kenya. The prevalence reported in this study was higher than the prevalence 36.5% (n= 236) and 45.0% (n= 100) reported by Leiva-Salinas [29] and Dogo et al. [30] from Ethiopia and Nigeria, respectively.

Prevalence of dermatophytosis according to gender of studied participants found in this study showed that males have the highest prevalence compared to the females. This was in line with finding of Leiva-Salinas et al. [29] who reported higher prevalence of dermatophytosis in males than in females 42.2% and 30.5%, respectively, among school children in a rural area in southern Ethiopia. This was contrary to the finding of Dogo et al. [30] who reported higher prevalence of dermatophytosis in girls than the boys 51.4% and 41.5%, respectively, among school children in Nok community of Kaduna State, Nigeria.

The prevalence of dermatophytosis was significantly (p=0.001) associated with age groups of studied participants which showed that age groups between 11-20 and 21-31 had the highest prevalence. This was in line with finding of Dogo et al. [30] who reported higher prevalence of dermatophytosis among age groups between 11 and 15 years among school children in Nok community of Kaduna State, Nigeria. This was contrary to the finding of Leiva-Salinas et al. [29] who reported higher prevalence of dermatophytosis 46.9% in age groups between 5 and 7 years among school children in a rural area in southern Ethiopia.

This study also revealed that out of the 49% samples that were positive by microscopy, only 28 samples were positive by culture. Wiegand et al. [9] reported 82.6% (n = 115) and 87.8% (n = 101) for Blankophor and culture methods, respectively. Thirty-one fungal species were isolated from the 28 samples positive by culture method which involved dermatophytes and non-dermatophytes. Among the dermatophytes species,* T. verrucosum *was the most common agent of dermatophytosis in this study. This was contrary to the finding of Wiegand et al. [9] who reported higher distribution of* T. violaceum *65.2 (73/112) among the dermatophytes species isolated from their study. However,* Aspergillus *spp. were found the most occurrent non-dermatophytes fungal species in this study. This was also contrary to the finding of Wiegand et al. [9] who reported higher prevalence of* Scopulariopsis brevicaulis* 6.3 (7/112) from their study.

The percentage yield of 10.05% was obtained from* T. riparia *ethanolic leaves crude extract and was within the recommended range (10.96-20.29%) according to the British Herbal Pharmacopoea [31]. This is also in line with the findings of [14].

Photochemistry of the plant revealed presence of tannins, steroids, glycosides, phenols, and terpenoids. This was similar to the finding of [15, 32] who reported the presence of reducing compounds, phenols and tannins from* T. riparia* ethanolic leaves crude extract. In addition, [33] reported the presence of saponins, flavonoids, and alkaloids from the* T. riparia* ethanolic leaves crude extract from Kenya. This confirmed the statement by [34] who reported that plant from different geographical area across the world may have different concentrations of the active substances.

Preliminary antifungal assay using modified agar well diffusion method revealed that* T. riparia* ethanolic leaves crude extract had antifungal activity against all tested dermatophytes at concentration of 1 g/ml, and terbinafine had antidermatophytic activity at a concentration of 40 *μ*g/ml. Endo et al. [35] also reported antidermatophytic activity of hydroalcoholic extract of* T. riparia* against some dermatophytes though they used disc diffusion method in their study. However, susceptibility of both terbinafine and extract varied among organisms with* T. tonsurans* being more susceptible followed by* T. mentagrophyte* while* M. audouinii* was less sensitive to the crude extract. The increased susceptibility to* T. tonsurans* and* T. mentagrophyte* could be attributed to their slow growth, i.e.,* T. tonsurans* (8-12 days) and* T. mentagrophyte* (7 -10days) as compared to* M. audouinii* which grows in 5-10 days [33]; hence the antifungal agent arrests their initial stages of growth, hence limiting their ability to sporulate thus them having increased susceptibility. Furthermore, terbinafine which was used as positive control showed higher antidermatophytic activity against all tested dermatophytes spp. compared to* T. riparia* leaves crude extract. This could be due to the purity of the terbinafine compared to the* T. riparia* leaves crude extract which was in mixture form. Ahmad and Aqil [34] reported that extracts in crude forms may contain some compound that may have antagonistic activity against other bioactive compounds in the crude extracts leading to the low activity against tested organism.

The MIC and MFC of terbinafine were also generally less for* T. tonsurans* (0.0078 and 0.0156 mg/ml), followed by* T. mentagrophyte* (0.0078 and 0.0313 mg/ml) while* M. audouinii* had 0.03 3 and 0.0625 mg/ml. This finding is similar to the findings of [36, 37] against similar organisms when using terbinafine. Terbinafine causes changes in the fungal cell permeability with consequent modification of the cell structure which also causes hyphae to become parched and wrinkled when viewed under an electron microscope. The variation in susceptibility patterns could be attributed to the growth period variations among the organisms [34].

## 5. Conclusions

The findings of this study reported the prevalence of dermatophytes causing dermatophytosis 49% among patients attending Dermatology and CHAI Clinics of KIU-TH. The prevalence of dermatophytosis was significantly (p=0.001) associated with age groups of studied participants.* T. riparia* leaves ethanolic crude extract contains bioactive compounds that are active against the tested dermatophytes* (T. tonsurans, T. mentagrophyte*, and* M. audouinii*). This supports its use in Western Ugandan traditional medicine for the treatment of skin diseases caused by dermatophytes and other fungal infections. This study found out the MIC range of the extract ranged from 62.5 to 250 mg/ml and MFC was 125-500 mg/ml. Further studies should be carried out on pure standard dermatophyte isolates included in the study and other dermatophyte and non-dermatophyte organisms not included in the study.

## Figures and Tables

**Table 1:** Prevalence of dermatophytosis among patients attending Kampala International Hospital based on microscopic examination (KOH) according to gender.

Gender	No. of samples collected	Microscopic (KOH)			Culture n=49		
		Positive	Negative	Total (%)	Positive	Negative	Total (%)
		n (%)	n (%)		n (%)	n (%)	
Male	53	26(49.06)	27 (50.94)	53(100)	19 (38.78)	7(14.29)	26 (53.06)
Female	47	23(48.93)	24 (51.06)	47(100)	09(18.37)	14(28.57)	23(4994)

*Total*	*100*	*49 (100)*	*51(100)*	*100(100)*	*28(57.15)*	*21(42.85)*	*49(100)*

Key: Microscopic results were not statistically significant using chi-square, *p = 0.990*.

**Table 2:** Prevalence of dermatophytosis among patients attending Kampala International Hospital based on microscopic examination (KOH) according to age.

Age	No. of samples collected	Microscopy (KOH)		Culture n =49	
		Positive, n (%)	Negative	Positive, n (%)	Negative
			n (%)		n (%)
1-10	3	3(100)	0 (0.00)	2(4.08)	1(2.04)
11-20	16	12(75.00)	4(25.00)	10(20.41)	2(4.08)
21-30	20	13(75.00)	7(35.00)	7(14.29)	6(12.24)
31-40	26	13(50.00)	13(50.00)	8(16.33)	5(10.20)
41-50	16	6(37.5)	10(62.5)	1(2.04)	5(10.20)
51-60	9	1(11.11)	8(88.89)	0(0.00)	1(2.04)
>60	10	1(10.00)	9(90.00)	0(0.00)	1(2.04)

*Total*	*100*	*49(49)*	*51 (51)*	*28 (57.14)*	*21* (42.86)
		*95*%*CI: 0.3930-0.5876*^*∗*^		*95*%*CI: 0.1987-0.3739*	

Key: Microscopic results were statistically significant for age group using chi-square, *p = 0.001*^*∗*^, CI: confidence interval.

**Table 3:** Macroscopic and microscopic characteristics of fungi isolated from patients attending Kampala International University Teaching Hospital, Western Campus, Uganda.

No.	Culture Morphology on SDA	Microscopic examination	Fungi identified
1.	The colonies were suede-like and few powdery with a flat, raised and folded centre with concentric furrow. The colour may vary from white, beige, grayish, and pale yellow pale-buff to yellow to dark-brown. The reverse appeared as yellow-brown to reddish-brown.	It had branched hyphae which were relatively wide and had numerous septa. It had numerous microconidia with varying sizes from long clavate to broad pyriform that were borne at right angles to the hyphae typical of “birds on a wire”. Very occasional few macroconidia were present on some cultures and appeared as smooth, thin-walled, irregular, and clavate shaped. Swollen giant forms of microconidia and chlamydospores were produced in older cultures.	*Trichophyton tonsurans*

2.	The colonies were flat, greyish-white to light tan-white in colour, and had a dense suede-like to downy surface that spread on entire culture. The reverse appeared yellow-brown to reddish-brown in colour.	Its hyphae were septate and exhibited a pectinate (comb-like) and racquet like structures with having thick-walled terminal or intercalary chlamydospores. The terminal chlamydospores were uniquely tapered at their apical end. Macroconidia and microconidia were not seen.	*Microsporum audouinii*

3.	The colonies had a powdery to granular surface and appeared white to cream in colour which gradually turned yellowish. A few cultures had raised central tufts with foldings. The reverse appeared yellow-brown then later reddish-brown in colour.	Its hyphae were septate and some were spiral shaped at the end. Few hyphae had chlamydospores that were spherically shaped. Numerous single celled microconidia that were subspherically shaped and few with clavate to pyriform were formed in clusters. Multicelled macroconidia that were thin-walled and had clavate-shape also were present.	*Trichophyton mentagrophytes*

4.	The colonies were flat and slightly raised, appeared white to cream, and became rose on aging and became suede-like to down; its reverse appeared yellow-brown to wine-red in colour.	It had a hyaline septate hyphae with numerous chlamydospores only in older cultures. Macroconidia were only seen in a few older cultures that appeared thin-walled multiseptate and slender cylindrical. It had numerous clavate to pyriform shaped microconidia.	*Trichophyton rubrum*

5.	The colonies appeared flat, with a dense cottony, fluffy-hairy surface having radiating furrow. Its obverse exhibited a white to cream colour with light yellowish pigment at its periphery. The reverse was pigmented with pale tan to yellowish which turned brownish on aging.	It had a septate hyphae with numerous macroconidia. The macroconidia were multicelled (6-14 cells), long, typically spindle-shaped, thick-walled with an echinulate texture. The terminal apex of the macroconidia was tapered into a knob like end. A few pyriform to clavate microconidia was rarely seen and appeared club shaped formed along the length of the hyphae.	*Microsporum canis*

6.	Colonies (SDA) are slow growing, small, button or disc-shaped, white to cream coloured, with a suede-like to velvety surface, a raised centre, and flat periphery with some submerged growth. Reverse pigment may vary from non-pigmented to yellow.	Chlamydospores are often in chains. The tips of some hyphae are broad and club-shaped, and occasionally divided, giving the so-called “antler” effect. *Trichophyton verrucosum *showing clavate to pyriform microconidia, characteristic rat tail or string bean-shaped macroconidia, terminal vesicles at the tips of hyphae in young colonies and chains of chlamydospores.	*Trichophyton verrucosum*

7.	The colonies appeared folded, glabrous, and mounded with deep violet in colour.	No conidia are usually seen. The hyphae were seen moderately broad with numerous branches. The hyphae from older cultures had significant number of chlamydospores present.	*Trichophyton violaceum*

8.	The colonies were fast growing, flat, granular, and few radial groves. The observe varied in colour from white, yellow, yellow-brown, brown to black, or dark yellow-green on aging. However, other colonies were classically unadorned green in colour. The reverse of each colony varied from greyish to purple-brown.	Conidial heads are short columnar and biseriate. Stipes are usually short, brownish, and smooth-walled. Conidia are globose and rough-walled. It had short biseriate conidial heads with flask shaped phialides borne on metulae. These were slightly spherical where conidia were produced. Conidia were globose (shperical) in shape.	*Aspergillus *spp

9.	The colonies were medium sized and appeared white to cream-coloured with a smooth, waxy, and glabrous surface with a yeast-like smell.	The yeast cells were spherical to subspherical in shape (budding blastoconidia) with a pseudohyphae. With India Ink Preparation, no capsules were present. It produced germ tubes when incubated in 0.5 mL of serum containing 0.5% glucose at 35°C for 2-3 hours.	*Candida albicans*

10.	The colonies were fast growing as pink with abundant cottony/hair like mycelium.	It had septate hyphae. Macroconidia were absent. Microconidia were spindle shaped, rounded, and tapered at each end, contained one/three cells. It had numerous intercalary chlamydospores which were ovoidal in shape while a few were ellipsoidal in shape.	*Fusarium *spp.

**Table 4:** Distribution of dermatophytes among patients attending Kampala International Hospital according to age and sex.

Demographic factors	Dermatophytes and other fungi associated with dermatomycosis n=31										Total (%)
	*M. canis *n (%)	*M. audouinii *n (%)	*T. violaceum *n (%)	T. tonsurans n (%)	T. verrucosum n (%)	*T. rubrum *n (%)	*T. mentagrophytes *n (%)	*Candida* spp. n (%)	*Aspergillus* spp. n (%)	*Fusarium *spp. n (%)	
*Age*											
1-10	0(0.00)	0(0.00)	0(0.00)	0(0.00)	4(12.90)	0(0.00)	0 (0.00)	0(0.00)	0(0.00)	0(0.00)	4 (12.90)
11-20	1(3.23)	1(3.23)	2(6.45)	0(0.00)	0(0.00)	1(3.23)	0(0.00)	0(0.00)	0(0.00)	0(0.00)	5 (16.13)
21-30	2 (6.45)	0(0.00)	0(0.00)	1(3.23)	1(3.23)	2(6.45)	0(0.00)	0(0.00)	1(3.23)	0(0.00)	7(22.58)
31-40	1(3.23)	0(0.00)	1(3.23)	0(0.00)	1(3.23)	1(3.23)	1(3.23)	0(0.00)	2(6.45)	1(3.23)	8(25.83)
41-50	1(3.23)	0(0.00)	0(0.00)	0(0.00)	0(0.00)	0(0.00)	0(0.00)	0(0.00)	2(6.45)	0(0.00)	3(9.68)
51-60	0(0.00)	0(0.00)	0(0.00)	0(0.00)	0(0.00)	0(0.00)	0(0.00)	1(3.23)	2(6.45)	0(0.00)	3(9.68)
>60	0(0.00)	0(0.00)	0(0.00)	0(0.00)	0(0.00)	0(0.00)	0(0.00)	1(3.23)	0(0.00)	0(0.00)	1(3.23)

*Total*	*5(16.13)*	*1 (3.23)*	*3(9.68)*	*1(3.23)*	*6 (19.35)*	*4(12.90)*	*1(3.23)*	*2(6.45)*	*7(22.58)*	*1(3.23)*	*31(100)*

*Sex*											
Male	1(3.23)	1(3.23)	3(9.68)	1(3.23)	4(12.90)	3(9.68)	0(0.00)	1(3.23)	6(19.35)	0(0.00)	20 (64.52)
Female	4(12.90)	0(0.00)	0(0.00)	0(0.00)	2(6.45)	1(3.23)	1(3.23)	1(3.23)	1(3.23)	1(3.23)	11(35.48)

*Total*	*5(16.13)*	*1(3.23)*	*3(9.68)*	*1(3.23)*	*6(19.35)*	*4(12.90)*	*1(3.23)*	*2(6.45)*	*7(22.58)*	*1(3.23)*	*31(100)*

**Table 5:** Qualitative phytochemical screening of *T. riparia* ethanolic leaves crude extract.

Phytochemical parameters	Inference
Saponins	-
Tannins	+
Phenols	+
Flavanoids	-
Glycosides	+
Alkaloids	-
Steroids	+
Terpenoids	+
Phlobatannins	-
Anthraquinones	-

Key: (+) indicates presence and (–) indicates absence.

**Table 6:** Minimum inhibitory concentrations and minimum fungicidal concentration.

Extract and Control	*Test Organisms*					
	*T. tonsurans*		*T. mentagrophyte*		*M. audouinii*	
	MIC (mg/ml)	MFC (mg/ml)	MIC (mg/ml)	MFC (mg/ml)	MIC (mg/ml)	MFC (mg/ml)
Plant Extract	62.5	125	125	250	250	500
Terbinafine	0.0078	0.0156	0.0078	0.0313	0.0313	0.0625

## Data Availability

The [Tables and Figures] data used to support the findings of this study are included within the article.
